# Supplementary material for: Detecting genomic deletions from high-throughput sequence data with unsupervised learning
Source: BMC Bioinformatics. 2023 Jan 27;23(Suppl 8):568. doi: 10.1186/s12859-023-05139-w (PMC9881243; doi:10.1186/s12859-023-05139-w)
Supplement: Supplementary file 1 — Additional file 1. Supplemental materials for “Calling genomic deletions from sequence data using unsupervised learning”. It contains the additional results presented in this paper. “A F1 score of NA12878 in each chromosome” contains the results of F1 score for each chromosome in NA12878 respectively. “B Command lines used for genomic deletions detection” is about the command lines used by each tool for deletion detection. [file 12859_2023_5139_MOESM1_ESM.pdf]

Supplemental materials for 'Detecting genomic deletions from high-throughput sequence data with unsupervised learning"

A F1 score of NA12878 in each chromosome

Method	Chr1	Chr2	Chr3	Chr4	Chr5	Chr6	Chr7	Chr8	Chr9	Chr10	Chr11	Chr12
EigenDel	0.53	0.56	0.52	0.63	0.52	0.59	0.49	0.55	0.59	0.53	0.58	0.55
Delly	0.48	0.51	0.50	0.50	0.45	0.52	0.46	0.56	0.51	0.50	0.58	0.54
Lumpy	0.47	0.52	0.55	0.56	0.56	0.55	0.39	0.54	0.58	0.58	0.56	0.63
Pindel	0.003	0.004	0.004	0.005	0.005	0.005	0.004	0.004	0.004	0.004	0.005	0.003
CNVnator	0.10	0.15	0.14	0.07	0.11	0.10	0.11	0.10	0.04	0.03	0.08	0.12
GASVpro	0.27	0.31	0.28	0.34	0.34	0.34	0.26	0.34	0.31	0.30	0.33	0.35
SvABA	0.002	0.002	0.002	0.003	0.005	0.002	0.003	0.004	0.002	0.001	0.004	0.002
Manta	0.078	0.116	0.1	0.083	0.091	0.101	0.093	0.089	0.108	0.092	0.095	0.07

Method	Chr13	Chr14	Chr15	Chr16	Chr17	Chr18	Chr19	Chr20	Chr21	Chr22	ChrX
EigenDel	0.52	0.57	0.51	0.55	0.45	0.60	0.52	0.43	0.33	0.46	0.34
Delly	0.54	0.52	0.54	0.44	0.38	0.58	0.46	0.49	0.47	0.50	0.33
Lumpy	0.54	0.50	0.62	0.55	0.43	0.62	0.49	0.43	0.47	0.36	0.39
Pindel	0.005	0.004	0.003	0.004	0.002	0.005	0.003	0.004	0.002	0.005	0.004
CNVnator	0.04	0.12	0.04	0.07	0.10	0.09	0.07	0.04	0.003	0.05	0.06
GASVpro	0.31	0.31	0.36	0.23	0.27	0.31	0.28	0.25	0.22	0.30	0.21
SvABA	0.001	0.006	0	0.004	0.002	0.003	0.003	0	0	0.004	0.005
Manta	0.091	0.086	0.11	0.12	0.044	0.098	0.082	0.07	0.038	0.106	0.105

Table S1. F1 score of NA12878 in each chromosome.

B Command lines used for genomic deletions detection

B.1 CNVnator

#!/bin/bash
#SBATCH -n 24
#SBATCH -N 1
#load the pre-required modules
module load gcc/5.4.0-alt zlib/1.2.11 java/1.8.0_162 mpi/openmpi/3.1.3 samtools/1.3 openssl/1.0.2o libcurl/7.60.0 git/2.7.2 xz/5.2.2-gcc540 bzip2/1.0.6 root/2.1
CNVnator=/scratch/xil14026/sv/software/CNVnator/0.4/cnvnator
ROOT=/scratch/xil14026/sv/data/1000Genome
BAMFILE=$ROOT/NA12763.mapped.ILLUMINA.bwa.CEU.low_coverage.20130502.bam
REFDIR=$ROOT/phase3_sv/ref_each_chrom/ref_name_std
BINSIZE=500

```
#Do the main logic of CNVnator
# 1: Extract read mapping
echo ”Step 1: Extract read mapping”
$CNVnator -root file.root -tree $BAMFILE
# 2: Generate histogram
echo ”Step 2: Generate histogram”
$CNVnator -root file.root -his $BINSIZE -chrom 1 2 3 4 5 6 7 8 9 10 11 12
13 14 15 16 17 18 19 20 21 22 X Y
# 3: Calculate statistics
echo ”Step 3: Calculate statistics”
$CNVnator -root file.root -stat $BINSIZE -d $REFDIR/
# 4: Partition
echo ”Step 4: Partition”
$CNVnator -root file.root -partition $BINSIZE
# 5:Call CNVs
echo ”Step 5: Call CNVs”
$CNVnator -root file.root -call $BINSIZE
```

B.2 Delly

```
#!/bin/bash
#SBATCH -p general
#SBATCH -n 24
#SBATCH -N 1
module purge
module load gcc/5.4.0-alt xz/5.2.2-gcc540 bcftools/1.9 singularity/3.1
ROOT=/scratch/xil14026/sv/data/1000Genome
REF=$ROOT/phase3_sv/ref/hs37d5.fa
BamFile=$ROOT/NA12763.mapped.ILLUMINA.bwa.CEU.low_coverage.20130502.bam
DELLY=/scratch/xil14026/sv/software/delly/singularity
singularity exec $DELLY/delly_sing/ delly call -o delly.bcf -g $REF $BamFile
bcftools view ./delly.bcf > ./delly.vcf
awk ‘if($7 == “PASS”) print $0‘ ./delly.vcf > ./delly_pass.vcf
```

B.3 GASVpro

```
#!/bin/bash
#SBATCH -n 24
#SBATCH -N 1
#SBATCH -p general
module load java/1.8.0_31 ant/1.9.4
#Main logic
GASVPATH=/scratch/xil14026/sv/software/GASV/gasv/bin
SAMPLENAME=NA12763.mapped.ILLUMINA.bwa.CEU.low_coverage.20130502
BAMFILEFOLDER=/scratch/xil14026/sv/data/1000Genome
```

BAMFILE=$BAMFILEFOLDER/$SAMPLENAME.bam
#1: Using BAMToGASV to Preprocess BAM files
echo "Step 1: Using BAMToGASV to Preprocess BAM files"
java -Xms512m -Xmx2048m -jar
$GASVPATH/BAMToGASV.jar $BAMFILE -LIBRARY_SEPARATED all
#2: Call structure variation
java -jar $GASVPATH/GASV.jar –batch $BAMFILEFOLDER/$SAMPLENAME.bam.gasv.in

B.4 Lumpy

#!/bin/bash
#SBATCH -p HaswellPriority
#SBATCH -A maa13014
#SBATCH -n 24
#SBATCH -N 1
module purge module load speedseq/0.1.2 bwa/0.7.5a python/2.7.6
module load perl/5.24.1 gcc/5.4.0-alt samtools/1.3 zlib/1.2.11 openssl/1.0.2o libcurl/7.60.0 cmake/3.8.0 python/2.7.6 pre-module post-module intelics/2017 git/2.7.2
module unload zlib
module load zlib/1.2.8-ics lumpy
SAMPLE=NA12763
REF=/scratch/xil14026/sv/data/1000Genome/phase3_sv/ref/hs37d5.fa
ROOT=/scratch/xil14026/sv/data/1000Genome/phase3_sv/Samples/$SAMPLE/reads
READ1=$ROOT/$SAMPLE.PE.1.fastq
READ2=$ROOT/$SAMPLE.PE.2.fastq
#1: generate bam file (mapped, split bam and discordant)
speedseq align -R "@RG/tID:id/tSM:$SAMPLE/tLB:lib" -t 12 $REF $READ1 $READ2
#2:run lumpy
BAM=./$SAMPLE.PE.1.fastq.splitters.bam SPLITBAM=./$SAMPLE.PE.1.fastq.splitters.bam DISCORDBAM=./$SAMPLE.PE.1.fastq.discordants.bam
lumpyexpress -B $BAM -S $SPLITBAM -D $DISCORDBAM -o sample.vcf

B.5 Pindel

#!/bin/bash
#SBATCH -p general
#SBATCH -n 24
#SBATCH -N 1
module purge
module load samtools/1.3 zlib/1.2.11 openssl/1.0.2o libcurl/7.60.0 git/2.7.2 gcc/5.4.0-alt bwa/0.7.15 xz/5.2.2-gcc540 htslib/0.0.1 r/3.1.1 java/1.8.0_31 pdftk/2.02 bedtools/2.27.1-gcc540a
#Set variable for pindel

SAMPLE=NA12763
PINDEL=/scratch/xil14026/sv/software/pindel_new/pindel/pindel
REF=/scratch/xil14026/sv/data/1000Genome/phase3_sv/ref/hs37d5.fa
CHROM=ALL
PREFIX=./output/$SAMPLE
BAMCONFIG=./bam.config
THREAD=24
mkdir -p ./output
$PINDEL -f $REF -i $BAMCONFIG -c $CHROM -o $PREFIX -T $THREAD

B.6 EigenDel

#!/bin/bash
#SBATCH -p general
#SBATCH -n 24
#SBATCH -N 1
module purge
module load samtools/1.3 gcc/5.4.0-alt java/1.8.0_31 sqlite/3.18.0 tcl/8.6.6.8606 python/3.6.1 r/3.1.1
../../../../MakeFile/EigenDel ./config.ini

B.7 Manta

configManta.py - -bam ${strBAM} - -referenceFasta ${strRef} - -runDir ${strCurSampleDir}

B.8 SvABA

cd ${strCurSampleDir} && ${binSVABA} run -t ${strBAM} -p 12 -L 6 -I -a germline_run -G ${strRef}

B.9 Comments

For details please check:
https://github.com/lxwgcool/EigenDel/tree/master/HPC_Job_Script_SLURM_Example
